# Murine GRPR and Stathmin Control in Opposite Directions both Cued Fear Extinction and Neural Activities of the Amygdala and Prefrontal Cortex

**DOI:** 10.1371/journal.pone.0030942

**Published:** 2012-02-01

**Authors:** Guillaume Martel, Charles Hevi, Alexandra Wong, Ko Zushida, Shusaku Uchida, Gleb P. Shumyatsky

**Affiliations:** Department of Genetics, Rutgers University, Piscataway, New Jersey, United States of America; Radboud University, Netherlands

## Abstract

Extinction is an integral part of normal healthy fear responses, while it is compromised in several fear-related mental conditions in humans, such as post-traumatic stress disorder (PTSD). Although much research has recently been focused on fear extinction, its molecular and cellular underpinnings are still unclear. The development of animal models for extinction will greatly enhance our approaches to studying its neural circuits and the mechanisms involved. Here, we describe two gene-knockout mouse lines, one with impaired and another with enhanced extinction of learned fear. These mutant mice are based on fear memory-related genes, stathmin and gastrin-releasing peptide receptor (GRPR). Remarkably, both mutant lines showed changes in fear extinction to the cue but not to the context. We performed indirect imaging of neuronal activity on the second day of cued extinction, using immediate-early gene c-Fos. GRPR knockout mice extinguished slower (impaired extinction) than wildtype mice, which was accompanied by an increase in c-Fos activity in the basolateral amygdala and a decrease in the prefrontal cortex. By contrast, stathmin knockout mice extinguished faster (enhanced extinction) and showed a decrease in c-Fos activity in the basolateral amygdala and an increase in the prefrontal cortex. At the same time, c-Fos activity in the dentate gyrus was increased in both mutant lines. These experiments provide genetic evidence that the balance between neuronal activities of the amygdala and prefrontal cortex defines an impairment or facilitation of extinction to the cue while the hippocampus is involved in the context-specificity of extinction.

## Introduction

**Figure 1:**
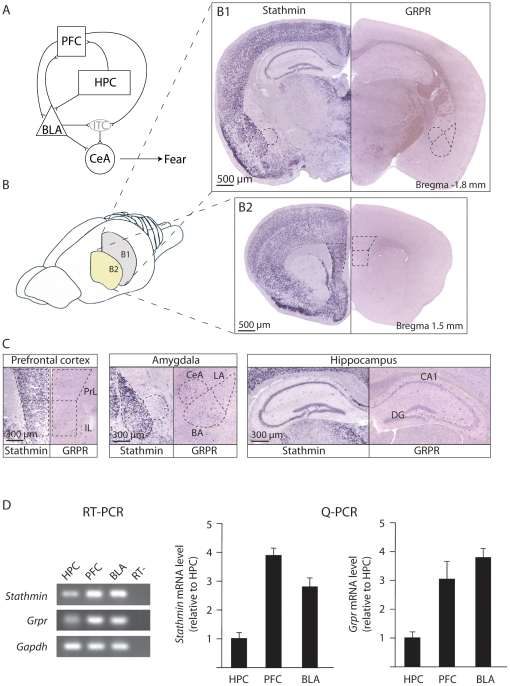
Expression of stathmin and GRPR in brain areas related to fear extinction. (**A**) Schematic representation of the connectivity of brain areas involved in fear extinction. (**B**) *In situ* hybridization of stathmin (left) and GRPR (right) in the hippocampus and amygdala (**B1**), and in the prefrontal cortex (**B2**). (**C**) High magnification pictures of stathmin and GRPR expression in the prefrontal cortex, amygdala and hippocampus. (D) Quantitative analysis of mRNA levels of *Stathmin* and *Grpr*. RT-PCR: Ethidium bromide stained gels of products of RT-PCR with cDNA isolated from the hippocampus (HPC), prefrontal cortex (PFC), and amygdala (BLA) tissues are shown. RT-, control PCR reaction without the reverse transcriptase step (to check for possible genomic DNA contamination). Q-PCR: The expression of S*tathmin* and *Grpr* mRNAs in the HPC, PFC, and BLA were quantified by Q-PCR (n = 4 for all groups). *Stathmin* and *Grpr* are expressed stronger in the PFC and BLA relative to HPC. PFC, prefrontal cortex; HPC, hippocampus; BLA, basolateral complex of the amygdala; CeA, central nucleus of the amygdala; ITC, intercalated nuclei, PrL, prelimbic division of prefrontal cortex; IL, infralimbic division of prefrontal cortex; BA, basal nucleus of amygdala; LA, lateral nucleus of the amygdala; DG, dentate gyrus; CA1, CA1 area of hippocampus.

Intensive research over the last several years has been focused on understanding the mechanisms involved in fear extinction, which is known to be impaired in several clinical conditions, such as post-traumatic stress disorder (PTSD) and panic disorder [1], [2], [3]. During extinction, an aversive conditioned stimulus (CS), previously paired with the unconditioned stimulus (US), gradually loses its ability to evoke the conditioned response (CR) after repeated presentations of the CS in the absence of the US, leading to a significant reduction in the CR. Importantly, cued extinction is context-specific, i.e., the CS still produces the CR in environments that are different from the extinction context. The ability of the CS after extinction to evoke the CR in the fear conditioning training context is termed renewal, suggesting that the original memory for the CS is not erased during the extinction. Indeed, much of the evidence demonstrates that extinction is an active learning process that produces inhibition over previous fear learning. It is thought that the amygdala is primarily involved in fear memory formation receiving inputs from the prefrontal cortex and hippocampus. Similarly, the basolateral amygdala is likely to be involved at the initial stages of extinction [4], [5] and the neural circuits of the amygdala, prefrontal cortex and hippocampus are all concerned with the inhibition of the original fear memory [6]. The connections between these structures are well described (Figure 1A) but their interaction and the balance between their neuronal activities are not clear. Similarly, the molecular mechanisms of extinction are not well understood [7]. Unraveling these aspects of fear extinction may have clinical implications, would require establishing animal models [3], [7], [8], and would greatly benefit from the use of genetically modified mice [9].

To examine how extinction is regulated by the balance of neural activities of the amygdala, prefrontal cortex, and hippocampus, we turned to two knockout (KO) mouse lines which, as we have previously reported, have opposing effects on fear memory and amygdala synaptic plasticity: gastrin-releasing peptide receptor (GRPR) KO mice have an increase whereas stathmin KO mice have a decrease in fear memory and synaptic plasticity relative to their wildtype controls [10], [11]. Here, we showed that these mutant mice have changes in opposite directions in fear extinction to the cue but not to the context compared to the control mice. Using c-Fos staining, we analyzed neuronal activity in the prefrontal cortex, amygdala, and hippocampus during extinction. Our results show that a shift in the balance of neuronal activity from the prefrontal cortex to the amygdala is accompanied by impaired extinction in GRPR KO mice; while a shift in the opposite direction (from the amygdala to the prefrontal cortex) is accompanied by extinction facilitation in stathmin KO mice.

## Results

### Expression of stathmin and GRPR in the amygdala, prefrontal cortex, and hippocampus

Both stathmin and GRPR were examined for their expression pattern using RNA *in situ* hybridization in three brain areas critically involved in fear extinction: the amygdala, hippocampus, and prefrontal cortex (Figure 1A). Previously, we and others showed that GRPR is located on inhibitory GABAergic interneurons in the amygdala [11], [12] and hippocampus [13], while stathmin is strongly expressed by the excitatory pyramidal cells [10]. *In situ* hybridization showed that stathmin was strongly expressed in the basolateral complex of the amygdala (BLA) which is comprised of the lateral (LA) and basal (BA) nuclei (Figures 1B1 and 1C), confirming our published data [10]. Stathmin had little expression in the central nucleus (CeA) and was not expressed in the intercalated nuclei (ITC). It was strongly expressed in the infralimbic (IL) and prelimbic (PrL) areas of the prefrontal cortex (Figures 1B2 and 1C). Stathmin was not expressed in the hippocampus except for a few cells in the dentate gyrus similar to the earlier observations by us and others {[10], [14]; Figures 1B1 and 1C and Figure S1; compare background staining on the brain slices from the KO mice in Figure S1A to staining on slices (examples of the stained cells are labeled with arrows) from the WT mice in Figure S1B}; there was little expression in the bed nucleus of stria terminalis (BNST) as we showed earlier ([15]; Figure S1C). GRPR was expressed in the LA, BA, and CeA in the amygdala as well as throughout the hippocampus [Note that GRPR is expressed by interneurons which are scattered throughout the brain and thus staining is not well visible with low magnification; Figures 1B1 and 1C and Figures S1D (KO mice) and S1E (WT mice)]. In the prefrontal cortex, GRPR was expressed by the infralimbic (IL) and prelimbic divisions (PrL; Figures 1B2 and 1C). There was no significant expression in the BNST (Figure S1F). We quantified mRNA levels of *Stathmin* and *Grpr* by RT/Q-PCR in wildtype mice (Figure 1D). For both *Stathmin* and *Grpr*, mRNAs were stronger expressed in the prefrontal cortex and BLA compared to the hippocampus [*Stathmin*: One-way ANOVA, F_(2,9)_ = 31.629, *P*<0.0001, Post-hoc (Scheffe's F); HPC vs PFC, *P*<0.001; HPC vs BLA, *P*<0.01, PFC vs BLA, *P*<0.05. *Grpr*: One-way ANOVA, F_(2,9)_ = 7.122, *P*<0.02, Post-hoc (Scheffe's F); HPC vs PFC, *P*<0.05; HPC vs BLA, *P*<0.05, PFC vs BLA, *P*>0.05]. These results demonstrate that both GRPR and stathmin are expressed in all these three brain areas involved in fear extinction.

### Fear extinction to the cue, but not to the context, is enhanced in stathmin KO mice and impaired in GRPR KO mice

**Figure 2:**
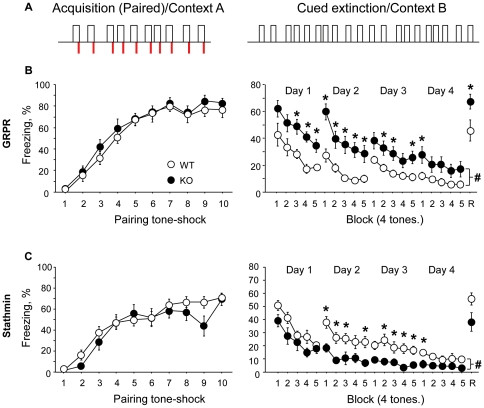
Cued fear extinction is controlled by stathmin and GRPR in opposite directions. (**A**) Protocol used for acquisition (left) and extinction (right) of cued fear conditioning. (**B**) Acquisition (left) and extinction (right) performances of GRPR WT and KO mice (11 WT and 12 KO). (**C**) Acquisition (left) and extinction (right) performances of stathmin WT and KO mice (16 WT and 11 KO). Acquisition performance is expressed as percentage of freezing during tone-shock pairings and extinction performance is expressed as percentage of freezing during 5 blocks (4 tones) for 4 days of extinction. Results are presented as mean ± SEM. R, renewal. *represents significant difference between groups during one block of a daily session; # represents significant difference between groups during the whole extinction phase.

We first examined GRPR KO mice and stathmin KO mice in cued (tone) fear extinction. During the acquisition phase (Figure 2A, left panel), both GRPR KO and WT mice learned the task (F_(9, 189)_ = 69.87; *P*<0.001) with a similar rate of progression between WT and KO mice (Figure 2B, left panel; *Ps*>0.51). At the beginning of extinction (Figure 2A, right panel), the GRPR WT mice froze to the tone 42% of the time and the GRPR KO mice froze 62%, but no significant difference between the genotype groups was detected (Figure 2B, right panel; *P*>0.074). Analysis of the first day with smaller bins (Figure S2A) confirmed that both genotypes expressed the same level of freezing during the first tone of the extinction session (*P*>0.118). Both groups extinguished through the four days of extinction (F_(19, 399)_ = 22.52; *P*<0.001) to reach almost the same level of freezing at the end of the fourth session (6% for GRPR WT mice and 17% for GRPR KO mice, *P*>0.089). A two-way ANOVA conducted on these data revealed a difference between the genotypes (F_(1, 21)_ = 12.42; *P*<0.002) with GRPR KO mice freezing more than their wild-type littermates. Analysis performed specifically for each day revealed that GRPR KO mice always froze more than their WT littermates (*Ps*<0.045). Additional analysis showed that the rate of extinction (decrease of freezing related to the initial freezing) was different only for day 2 (F_(1, 21)_ = 7.07; *P*<0.015) whereas on the other days a similar rate of extinction was observed (*P*>0.167). Fifteen days following the last day of the extinction phase, the mice were tested in the same context of acquisition to assess the initial memory (Figure 2B, right panel; R, renewal). During this test, GRPR KO mice froze more than their WT littermates. Student's t-test conducted on these data confirmed this observation (F_(1, 21)_ = 5.34; *P*<0.032).

Stathmin KO and WT mice learned the task during acquisition of cued fear conditioning (Figure 2C, left panel; F_(9, 225)_ = 35.88; *P*<0.001) with a similar rate of progression between the genotypes (*Ps*>0.27). At the beginning of extinction (Figure 2C, right panel), although stathmin WT mice froze 50% of the time to the tone and stathmin KO mice froze 40% of the time, no significant difference between the genotypes was detected (*P*>0.106). Further analysis of the first day of extinction (Figure S2B) confirmed that both genotypes displayed the same level of freezing during the first tone presentation (*P*>0.250). Both groups extinguished through the four days of the protocol (F_(19, 475)_ = 20.22; *P*<0.001) and reached different levels of freezing at the end of the fourth session (9.77% for stathmin WT mice and 2.57% for stathmin KO mice (F_(1, 25)_ = 5.88; *P*<0.023). A two-way ANOVA conducted on these data revealed a difference between the genotypes (F_(1, 25)_ = 6.05; *P*<0.022) with stathmin KO mice freezing less than their WT littermates. Both genotypes started at the same level of freezing but reached a different level of freezing at the end of the extinction protocol suggesting that stathmin KO mice had a faster rate of extinction than their WT counterparts. Analysis performed for each day separately revealed that stathmin KO mice froze less than their WT littermates only during day 3 and 4 (*Ps*<0.012) whereas no significant difference was detected during day 1 (*P*>0.129) and day 4 (*P*>0.058). Additional analysis showed that the rate of extinction (decrease of freezing related to the initial freezing) was different only for day 3 (F_(1, 25)_ = 5.04; *P*<0.040) whereas on the other a similar rate of extinction was observed (*Ps*>0.139). Fifteen days following the last day of the extinction phase, the mice were tested for renewal (Figure 2C, right panel; R, renewal). During this test both groups of mice froze at a similar level whereas a strong tendency of stathmin KO mice freezing less was observed. A Student's t-test conducted on these data showed no difference between the genotypes (*P*>0.068). Analysis of the progression between the end of the extinction session and the renewal revealed a significant difference between the genotypes (F_(1, 25)_ = 5.92; *P*<0.025).

Performances of WT animals in these experiments of cued fear conditioning were analyzed and no difference was detected between GRPR and stathmin WT animals in acquisition (*P*>0.141) or in extinction session (*P*>0.066).

**Figure 3:**
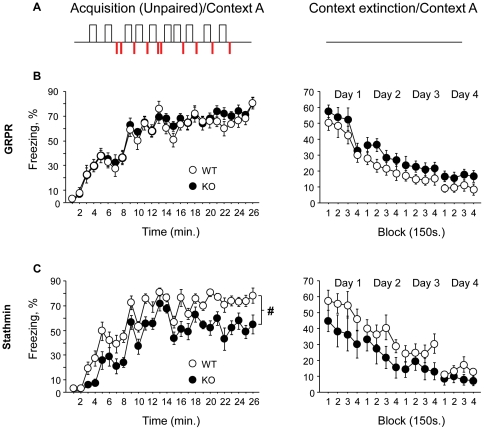
Stathmin and GRPR are not involved in contextual fear extinction. (**A**) Representation of the protocol used for acquisition (tone-shock explicitly unpaired, left) and extinction (right) of contextual fear conditioning. (**B**) Acquisition (left) and extinction (right) performances of GRPR WT and KO mice (12 WT and 12 KO). (**C**) Acquisition (left) and extinction (right) performances of stathmin WT and KO mice (11 WT and 11 KO). Acquisition performance is expressed as percentage of freezing minute by minute and extinction performance is expressed as percentage of freezing during 10 minutes of the session during 4 days of extinction. Results are presented as mean ± SEM. # represents significant difference between groups during the whole extinction phase.

In a separate experiment, we examined stathmin and GRPR KO mice in context fear extinction (Figure 3). During the acquisition phase (tone and shock explicitly unpaired; Figure 3A, left), both GRPR KO and WT mice learned the task (F_(25, 550)_ = 56.12; *P*<0.001) with a similar rate of progression between the genotypes (Figure 3B, left panel; *P*>0.516). At the beginning of extinction (Figure 3A, right panel), both genotypes froze at the same level (*P*>0.331) and extinguished through the four days of extinction (F_(15, 330)_ = 32.49; *P*<0.001) with a similar rate of progression between WT and KO mice (Figure 3B, right panel; *P*>0.090). Analysis performed separately for each day confirmed this description of the results showing that GRPR KO and WT mice expressed the same level of freezing through all extinction sessions (*Ps*>0.114).

During the acquisition phase (Figure 3A, left), both stathmin KO and WT mice learned the task (F_(25, 500)_ = 54.21; *P*<0.001) but with a different rate of progression between WT and KO mice (Figure 3C, left panel; F_(1, 20)_ = 14.32; *P*<0.002). Stathmin WT mice reached 78% of freezing at the end of the acquisition whereas stathmin KO mice reached only 55% (F_(1, 20)_ = 5.61; *P*<0.028). Surprisingly, at the beginning of extinction (Figure 3A, right panel), both stathmin KO and WT mice froze at the same level (*P*>0.18) and both groups extinguished through the four days of extinction (F_(15, 300)_ = 23.62; *P*<0.001) with a similar rate of progression between the genotypes (Figure 3C, right panel; *P*>0.135). Analysis performed separately for each day confirmed the absence of differences between stathmin KO and WT mice during all extinction sessions (*Ps*>0.126).

Performances of WT animals were compared in these experiments of context fear conditioning and no difference was detected between GRPR and stathmin WT mice either in acquisition (*P*>0.128) or in extinction (*P*>0.081).

### Brain activity during extinction

**Figure 4:**
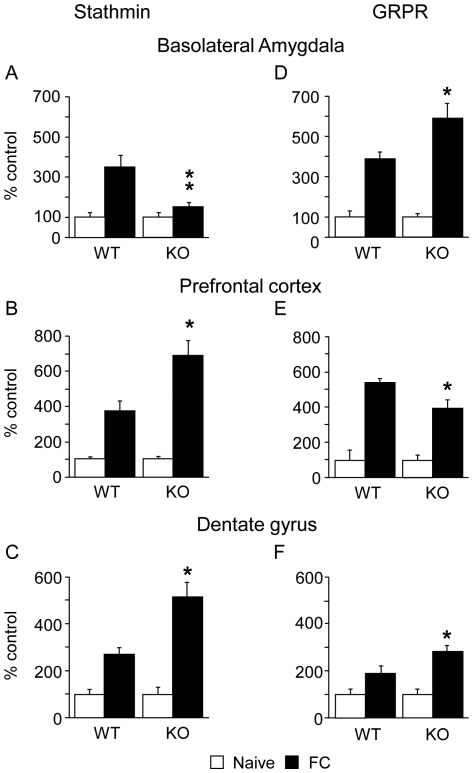
c-Fos is induced in opposite directions in stathmin KO and GRPR KO mice during extinction of cued fear conditioning. (**A**) and (**D**), c-Fos induction is decreased in the basolateral amygdala of stathmin KO mice (5 WT and 5 KO) whereas it is increased in GRPR KO mice (6 WT and 6 KO) compared to WT mice. (**B**) and (**E**) c-Fos induction is increased in the prefrontal cortex of stathmin KO mice whereas it is decreased in GRPR KO mice compared to WT mice. (**C**) and (**F**), c-Fos induction is increased in the dentate gyrus of both stathmin and GRPR KO mice compared to WT mice. Results are presented as mean ± SEM. *, P<0.05; **, P<0.01, compared to WT mice.

We analyzed brain activity on the second day of extinction because the difference on that day was the largest between WT and KO mice for both knockout lines. To examine the activity of the anatomic areas involved in fear extinction we used immediate-early gene c-Fos as a marker of neuronal activity (Figures 4 and S3-S4). c-Fos was induced in all anatomic areas involved in fear extinction (amygdala, hippocampus and prefrontal cortex) in all experimental groups compared to naïve mice (*Ps*<0.005). c-Fos induction was significantly lower in the basolateral amygdala (BLA) of stathmin KO mice compared to their WT littermates (Figure 4A; F_(1, 15)_ = 8.97; *P*<0.010; Scheffe's F, *P* = 0.0113) while c-Fos induction was significantly higher in the BLA of GRPR KO compared to their WT littermates (Figure 4D; F_(1, 20)_ = 5.36; *P*<0.032; Scheffe's F, *P* = 0.0307). In the central amygdala (Figures S3A and S3C) as well as in the CA1 (Figures S3B and S3D), c-Fos induction was similar between WT and KO for both stathmin (*Ps*>0.422) and GRPR mice (*Ps*>0.252). However, in the prefrontal cortex c-Fos induction was significantly higher in stathmin KO mice compared to their WT littermates (Figure 4B; F_(1, 16)_ = 8.97; *P*<0.010; Scheffe's F, *P* = 0.0143) whereas c-Fos induction was significantly lower in GRPR KO compared to their WT littermates (Figure 4E; F_(1, 20)_ = 3.06; *P*<0.010; Scheffe's F, *P* = 0.0218). Interestingly, in the dentate gyrus c-Fos induction was significantly higher in stathmin KO mice compared to their WT littermates (Figure 4C; F_(1, 16)_ = 11.04; *P*<0.010; Scheffe's F, *P* = 0.0053) as well as in GRPR KO compared to their WT littermates (Figure 4F; F_(1, 20)_ = 3.51; *P*<0.076; Scheffe's F, *P* = 0.0398).

## Discussion

**Figure 5:**
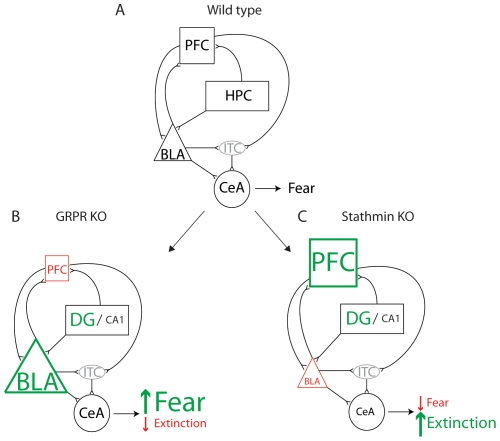
Schematic representation of the connectivity of brain areas involved in fear extinction. There is a balance between the amygdala, hippocampus and prefrontal cortex during normal fear reaction in wildtype mice (**A**). In GRPR KO mice there is a shift of the balance between the basolateral amygdala and prefrontal cortex towards stronger activation of the basolateral amygdala leading to higher freezing (**B**). Stronger neural activity in the prefrontal cortex leads and lesser in the basolateral amygdala leads to less freezing in stathmin KO mice (**C**).

In this study we investigated the roles of stathmin and gastrin-releasing peptide receptor in extinction of fear memory. We found that stathmin KO and GRPR KO mice have abnormal extinction of fear memory to the tone compared to their wildtype littermates (Figure 5A). Impaired cued extinction in GRPR KO mice was accompanied by stronger induction of neuronal activity (measured by c-Fos staining) in the basolateral amygdala and weaker induction in the prefrontal cortex compared to wildtype controls (Figure 5B). In contrast, stathmin KO mice demonstrated accelerated extinction, lesser induction of neuronal activity in the basolateral amygdala and stronger induction in the prefrontal cortex (Figure 5C). Taken together, our experiments suggest that the balance between the prefrontal cortex and amygdala is tightly correlated with the outcome of extinction.

The differential regulation of cued versus contextual fear extinction cannot be related to some difference in pain sensitivity or locomotor activity since both lines showed normal reaction to the pain and have normal locomotor activity [10], [11]. On the other hand, whereas GRPR KO mice have normal anxiety, stathmin KO mice showed an impairment of innate fear, which could be in relation with the deficit observed during the acquisition of contextual fear conditioning. This impairment in acquisition had no consequence on the contextual fear extinction since the mutant mice behave similarly to WT mice. Remarkably, fear extinction to the context was not affected in both mutant lines, although our previous work showed that contextual fear memory was enhanced in GRPR KO mice and decreased in stathmin KO mice [10], [11]. This may be a reflection of the single CS-US pairing protocol used in our earlier papers while in the current work we used ten CS-US pairings. There are very few reports that demonstrate differential regulation of cued versus contextual fear extinction. Virus-mediated overexpression of cAMP/Ca^2+^ responsive element binding protein (CREB) in the auditory thalamus specifically enhanced cued but not contextual fear memory [16]. Similarly, GRPR and stathmin are strongly expressed in the auditory thalamus which might lead to cued extinction specificity. To our knowledge, the only previously reported genetically modified mouse line which has impaired fear extinction to the cue but not to the context is the one with a global deletion of glutamic acid decarboxylase (GAD65) [17]. The authors of this report attributed the deficit in extinction to the deficiency in GABA function. Both GRPR KO mice and GAD65 KO mice have enhanced fear memory due to decreased GABA function. However, an important difference between the two knockout lines is that GAD65 KO mice have enhanced both innate fear/anxiety and learned fear [18], [19], [20], while the GRPR KO mice have an enhancement specific to learned fear [11]. The GRPR-positive interneurons represent approximately 10% of all interneurons in the amygdala [11]. It is tempting to speculate that some of the fear memory enhancement/fear extinction impairment phenotype found in GAD65 KO mice is based on malfunction of the GRPR positive interneurons. Previous results from our laboratory and others showed that GRPR is expressed almost exclusively on interneurons [11], . GRPR KO mice have a lack of inhibitory control of principal cells by GRPR-positive interneurons which results in a stronger memory for fear [11]. Thus, prolonged cued extinction in GRPR KO mice can result from the lack of inhibitory control failing to properly suppress the original memory of the CS-US association similar to GAD65 KO mice or GABA receptor alpha5 mutant mice, which have a reduction of GABAergic function and are resistant to extinction as well [21].

However, accelerated fear extinction in stathmin KO mice is likely to be dependent on a mechanism other than GABA function since we showed previously that GABA function is normal in stathmin KO mice and stathmin is expressed by excitatory principal neurons [10]. Stathmin KO mice have a deficit in learned fear and it is possible that an improvement in extinction is based on the ability of the new CS-noUS association to suppress the relatively weak original fearful CS-US association. Thus, more than just GABA-dependent mechanisms may be involved in cued extinction. According to currently prevalent theories, extinction does not result from a temporal decay or degradation of the original excitatory memory trace but is the result of an inhibition, which is formed during re-exposure to the CS in the absence of the US. This inhibitory memory trace (CS–no US) competes with and suppresses the original excitatory CS–US memory trace. This notion is supported by spontaneous recovery of the fear response with the passage of time after completion of extinction training [1], [22] and its renewal as observed in our experiments showing that 15 days after extinction the initial fear memory to the cue is intact when tested in the training context.

Changes in neural activity in the amygdala in both mutant lines are in agreement with our earlier work showing that GRPR KO mice have enhanced long-term potentiation (LTP) in the lateral amygdala and enhanced learned fear while stathmin KO mice have decreased amygdala LTP and deficient learned fear [10], [11]. We examined neuronal activity in the middle of the extinction session (after 10 tone presentations) of the second day because the difference in freezing for WT and KO mice was the greatest at the beginning of the extinction session on day 2 for both knockout lines. These earlier time points can be looked at as a recall of fear extinction learning from a previous day of extinction. In addition, a recent work that carefully examined extinction within a session versus extinction between sessions suggested that the earlier time points during extinction sessions are better predictors of extinction effectiveness [23]. These authors showed that within-session extinction is neither sufficient nor essential for between-sessions extinction, which is a more reliable indicator of long-term extinction. GRPR and stathmin KO mice are relatively similar to the WT controls in within-session extinction. However, in between-sessions extinction these lines of mice behave opposite to each other and are very different from the control mice. GRPR KO mice display a slow rate, while stathmin KO mice have a fast rate of between-sessions extinction.

The two opposite extinction phenotypes of GRPR KO and stathmin KO mice were paralleled by neural activities in the amygdala and prefrontal cortex. Stathmin KO mice had a lower expression of c-Fos in the basolateral amygdala whereas a higher neural activity was detected in the pre- and infralimbic cortex. GRPR KO mice presented an opposite pattern, i.e., stronger neural activity in the basolateral amygdala and a lower neural activity in the prefrontal cortex. Activity in the basolateral amygdala is thought to be associated with the level of fear expression, while activity in the prefrontal cortex with fear extinction [6]. Similarly, our present data show that low activity in the prefrontal cortex is correlated with slow extinction (GRPR KO mice) whereas high activity in the prefrontal cortex is correlated with fast extinction, which is consistent with the findings demonstrating that activation of the mPFC enhances extinction [24]. Recent work dissociated the roles of the infra- versus prelimbic cortices using local inactivation of muscimol before or after the extinction training [6]. These authors showed that infralimbic cortex was involved in the processes of extinction whereas the prelimbic cortex was involved in fear expression. Our experiments using c-Fos did not revealed such dissociation which can be explained by differences in our behavioral protocols and time points used to examine c-Fos activity. Similar to our c-Fos results, Knapska and Maren [25] found that the medial prefrontal cortex and dentate gyrus are activated during CS presentation in the extinction context. Again, in contrast to their results, we were unable to see differential activity in the infralimbic versus prelimbic divisions of the prefrontal cortex. This can be explained by different extinction protocols used by our and their labs in evaluation of c-Fos activity as well as by using rats in their work while our experiments employed mice.

In addition, our data suggest that the original memory for the CS in its original training context as well as in other contexts is very strong. Renewal experiments performed 15 days following 4 days of extensive extinction showed that the CS presented in the training context A elicited level of freezing that was no different from freezing elicited by the same CS at the beginning of extinction in context B. These data also suggest that storage and recall of the CS is normal in both mutants and their differences in fear memory are due to the initial learning and/or encoding.

In the current work, we assumed that GRPR and stathmin are not interacting biochemically because stathmin is predominantly expressed in the principal neurons and GRPR is almost exclusively in interneurons; therefore they do not co-localize in the same cells. However, cDNAs for the GRP neuropeptide and stathmin were identified in the same single cell cDNA library isolated from the amygdala pyramidal cell [11], which suggests that GRP neuropeptide (but not the GRP receptor) and stathmin are co-localized in a certain population of the excitatory neurons. Our earlier work suggested that some of the GRPR-positive interneurons have their local targets on the GRP-expressing pyramidal neurons, thus providing a negative feedback to the GRPergic excitatory cells in the basolateral amygdala [11]. It is possible that stathmin-expressing principal neurons release GRP neuropeptide, which can be co-released with glutamate during fear learning or extinction. In turn, the GRPR-positive interneurons are likely to inhibit both stathmin- and GRP-positive principal cells by GABA release. In the future it would be interesting to examine whether the cell signaling pathways involving GRP, GRPR and stathmin interact at the intracellular or intercellular level during fear learning or extinction.

Research on genetic animal models showing impaired or enhanced extinction can help our understanding of the molecular and cellular mechanisms of fear states in humans and would allow for direct comparisons of the extinction mechanisms between the two species [26]. Interestingly, recent work showed that stathmin mutations are involved in anxiety and fear in humans [27], thus a possibility exists that modeling fear extinction in mice may provide us with answers on how stathmin and other genes control similar extinction processes in humans.

## Methods

### Animals

Stathmin KO and GRPR KO were maintained on C57BL/6J background (N>10). The homozygous KO mice and their WT littermates were generated by breeding heterozygous pairs, which resulted from breeding of heterozygous mice to C57BL/6J mice (Jackson Laboratory). All mice were maintained on a 12 h light/dark cycle. Behavioral experiments were conducted during the light phase of the cycle, and mice were at least 12 weeks old at the time of training. This study was carried out in strict accordance with the recommendations in the Guide for the Care and Use of Laboratory Animals of the National Institutes of Health. The Rutgers University Institutional Animal Care and Use Committee approved the protocol. Rutgers University maintains an Assurance with the Office of Laboratory Animal Welfare, the assurance number is A3262-01.

### RNA isolation and cDNA synthesis

The hippocampus, medial prefrontal cortex (mPFC), and amygdala were dissected as previously reported [28], [29]. In brief, mouse brains were immediately extracted and put on ice. Bilateral punches (18 gauge) of the ventral area of the mPFC (including prelimbic and infralimbic subregions) and amygdala (preferentially including BLA) were obtained. Collected tissue was immediately frozen, and stored at −80°C until processing. Total RNA from dissected tissues was extracted by using the TRIzol Reagent (Invitrogen) and treated with DNase (Ambion). One microgram of total RNA was used for cDNA synthesis by TaqMan Reverse Transcription Reagents (Applied Biosystems). The cDNA was stored at −80°C until use.

### Quantitative real-time polymerase chain reaction (Q-PCR) and reverse transcription-PCR (RT-PCR)

Q-PCR was performed in the Applied Biosystems 7900HT Sequence Detection System with SYBR green PCR master mix (Applied Biosystems) according to the manufacturer's protocol. PCR conditions were 10 min at 95°C, 40 cycles of 15 s at 95°C and 60 s at 60°C. Amplification of the single PCR product was confirmed by monitoring the dissociation curve and electrophoresis on 1.2% agarose gels stained with ethidium bromide. Amplification curves were visually inspected to set a suitable baseline range and threshold level. The relative quantification method was employed for quantifying the amounts of target molecules according to the manufacturer's protocol, in which the ratio between the amount of target molecule and a reference molecule within the same sample was calculated. All measurements were performed in triplicate and four mice were used in each group. Levels of GAPDH mRNA was used to normalize the relative expression levels of target mRNA. RT-PCR was performed using Ex-Taq DNA polymerase (Takara), as previously reported [30]. PCR conditions were 4 min at 95°C, 30 cycles of 30 s at 95°C, 30 s at 56°C, and 30 s at 72°C. Amplification of the single PCR product was visualized by electrophoresis on 1.5% agarose gels stained with ethidium bromide. To control for genomic DNA contamination, primers were designed to span intron sequences, and RT were performed in the absence of Superscript (RT-). The PCR primers used in Q-PCR and RT-PCR were as follows (5′ to 3′): *Stathmin* forward, GTTCGACATGGCATCTTCTGAT; *Stathmin* reverse, CTCAAAAGCCTGGCCTGAA; *Grpr* forward, AATCTTCCCGTGGAAGGCAAT; *Grpr* reverse, TACTGTCTTGGCAAGCCGCTT; *Gapdh* forward, CTCCACTCACGGCAAATTCAA; *Gapdh* reverse, GATGACAAGCTTCCCATTCTCG.

### Immunohistochemistry

Twenty stathmin male mice (10 WT and 10 KO) and twenty four GRPR male mice (12 WT and 12 KO), 2–3 months old, were used. Half of the were naïve and the other half were subjected to the acquisition of the cued fear conditioning procedure, the first session of extinction and the first 10 tones of the second session of extinction. One hour after the completion of the first 10 tones of the second session of extinction, mice were perfused transcardially with ice-cold solution of 4% paraformaldehyde in phosphate buffer. After post-fixation overnight in the same fixative a 4°C, coronal sections (40 µm) were cut on a vibratome and collected in phosphate buffer.

After elimination of endogenous peroxydase activity and pre-incubation step, sections were incubated for 24 h with rabbit anti-Fos antibody (1∶10,000 dilution, Calbiochem). Subsequently, sections were incubated with biotinylated goat anti-rabbit antibody (1∶600; Vector) and with the ABC complex (ABC kit, Vector), and staining was visualized with diaminobenzidine (DAB, Sigma). Sections were mounted on gelatin-coated slides, air dried, dehydrated, covered with a glass coverslip using Eukitt (Fluka) mounting media and examined under light microscopy.

The number of c-fos-positive cells was counted bilaterally in the prefrontal cortex (an average of 3–4 sections per animal), in the hippocampus (CA1 and DG, an average of 5–6 sections per animal) and in the amygdala (an average of 4–5 sections per animal) using Image Pro plus 7 (Mediacybernetics). Counting was performed at 63× magnification. The anteroposterior (AP) coordinates relative to bregma of the areas included for detailed analyses were for the prefrontal cortex AP 1.54 to 1.98 mm, for the hippocampus AP-1.46 to −2.18 mm and for the amygdala AP −1.06 to −2.06 mm [25], [31].

### Behavior

All experiments were performed between 12 p.m and 7 p.m during the light phase of the day. To make sure that both WT and KO groups will freeze at the same level at the beginning of extinction, animals received a strong conditioning protocol and extinction started 5 hours after the end of the acquisition.

#### Extinction of cued fear conditioning

During the acquisition phase, GRPR mice (11 WT and 12 KO) and stathmin mice (16 WT and 11 KO) received ten paired presentations of tone CS (30 s, 2.8 kHz, 85 db) and shock US (2 s, 0.7 mA) trials with an average of 75-s inter-trial interval (ITI). The extinction phase started in a novel context 5 h following acquisition. The mice were exposed to twenty CS tones (34-min test) with an average of 80-s ITI each day for four consecutive days. Fifteen days following the end of the fourth session of extinction the initial memory (renewal) was assessed during presentation of the tone in the acquisition context.

#### Extinction of contextual fear conditioning

During context fear extinction training, GRPR mice (12 WT and 12 KO) and stathmin mice (11 WT and 11 KO) received ten shocks (2 s, 0.7 mA) trials with an average of 75-s ITI unpaired with ten tones (30 s, 2.8 kHz, 85 db). The extinction of fear memory started 5 h after fear conditioning in the same context as in acquisition. The mice were exposed 10 min to the context of acquisition during 4 consecutive days. Percentage of time spent freezing was measured by FreezeView software (Coulbourn Instruments).

### Statistical Analysis

Statistical analyses were run using Statview (SAS institute). Behavioral analyses were performed using one-way or two-way ANOVAs and Student's t-test. Post-hoc tests were performed using Scheffe's F.

## Supporting Information

Figure S1 **RNA** ***in situ*** **hybridization for** ***Stathmin*** **and** ***Grpr*** **in the hippocampus and BNST.** (A) Example of the background staining for the *Stathmin* dig-RNA probe on the hippocampal brain section from the stathmin KO mouse. (B) Example of stathmin-positive cells in the dentate gyrus (arrows) using the hippocampal brain section from the WT mouse. (C) The BNST has very little expression of stathmin. (D) Hippocampal brain sections from GRPR KO mouse have no *Grpr* RNA expression. (E) *Grpr* RNA strongly labels scattered cells (arrows) throughout the hippocampus in WT mice. (F) *Grpr* is not expressed in the BNST.
(TIF)

Figure S2 **Analysis of the first day of cued extinction revealed that GRPR and stathmin KO mice started at the same level.** (**A**) Percentage of freezing of GRPR KO mice during the first day of cued extinction. (**B**) Percentage of freezing of stathmin KO mice during the first day of cued extinction. Results are presented as mean ± SEM.
(TIF)

Figure S3 **No difference in c-Fos induction in the central amygdala and CA1 area of hippocampus during extinction in stathmin KO and GRPR KO mice compared to wildtype controls.** (**A**) and (**C**), c-Fos induction is the same in the central amygdala of stathmin KO and GRPR KO mice compared to their WT littermates. (**B**) and (**D**), c-Fos induction is the same in the CA1 hippocampal area of stathmin KO and GRPR KO mice compared to their WT littermates. Results are presented as mean ± SEM.
(TIF)

Figure S4 **Representative photographs of c-Fos staining during extinction.** c-Fos expression in the amygdala (**A**), prefrontal cortex (**B**) and hippocampus (**C**) of stathmin and GRPR WT and KO mice.
(TIF)
