# Supplementary figures and images for: Murine GRPR and Stathmin Control in Opposite Directions both Cued Fear Extinction and Neural Activities of the Amygdala and Prefrontal Cortex
Source: PLoS One. 2012 Feb 1;7(2):e30942. doi: 10.1371/journal.pone.0030942 (PMC3270024; doi:10.1371/journal.pone.0030942)

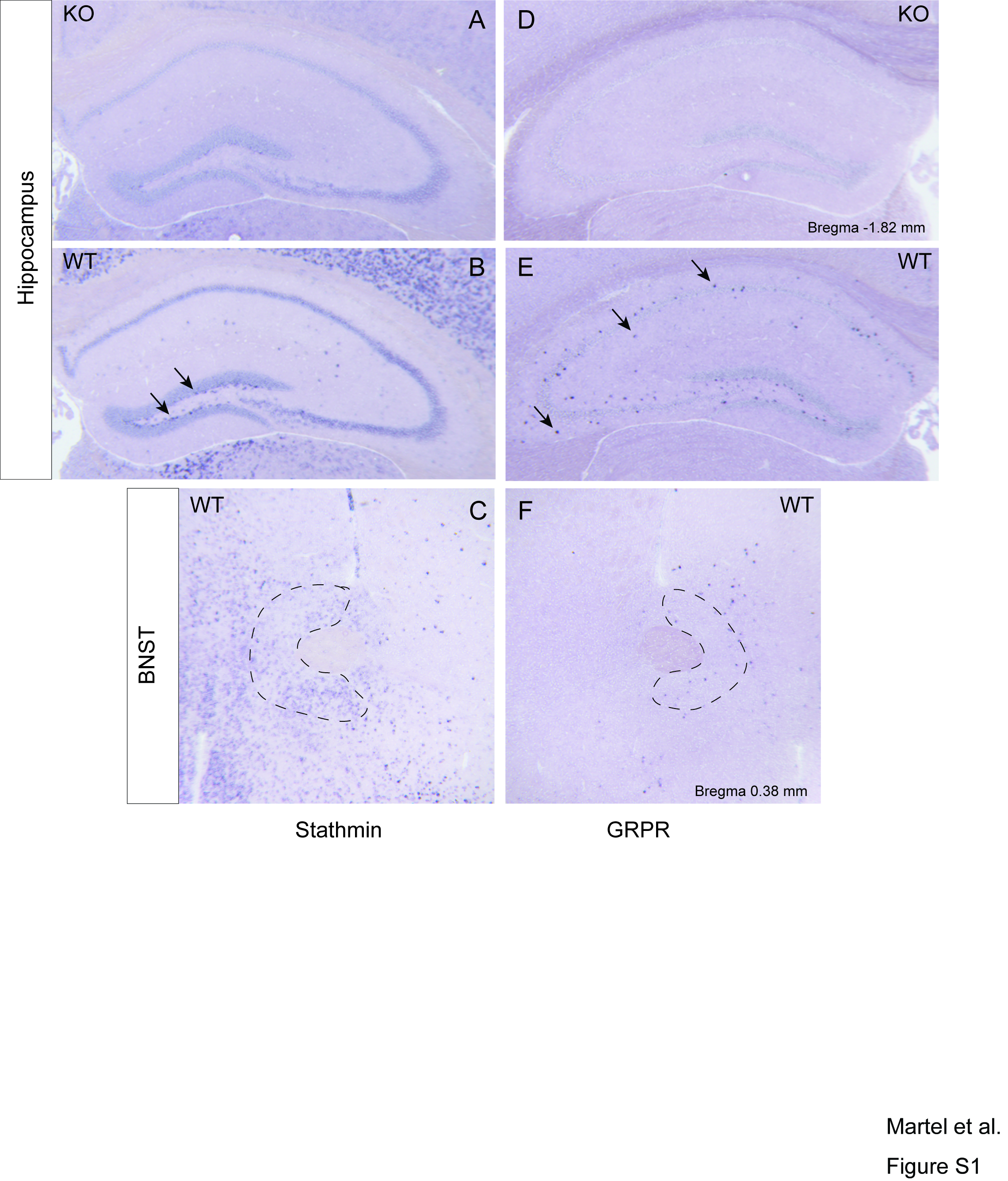

Supplement: Figure S1 — RNA in situ hybridization for Stathmin and Grpr in the hippocampus and BNST. (A) Example of the background staining for the Stathmin dig-RNA probe on the hippocampal brain section from the stathmin KO mouse. (B) Example of stathmin-positive cells in the dentate gyrus (arrows) using the hippocampal brain section from the WT mouse. (C) The BNST has very little expression of stathmin. (D) Hippocampal brain sections from GRPR KO mouse have no Grpr RNA expression. (E) Grpr RNA strongly labels scattered cells (arrows) throughout the hippocampus in WT mice. (F) Grpr is not expressed in the BNST. (TIF) [file pone.0030942.s001.tif]

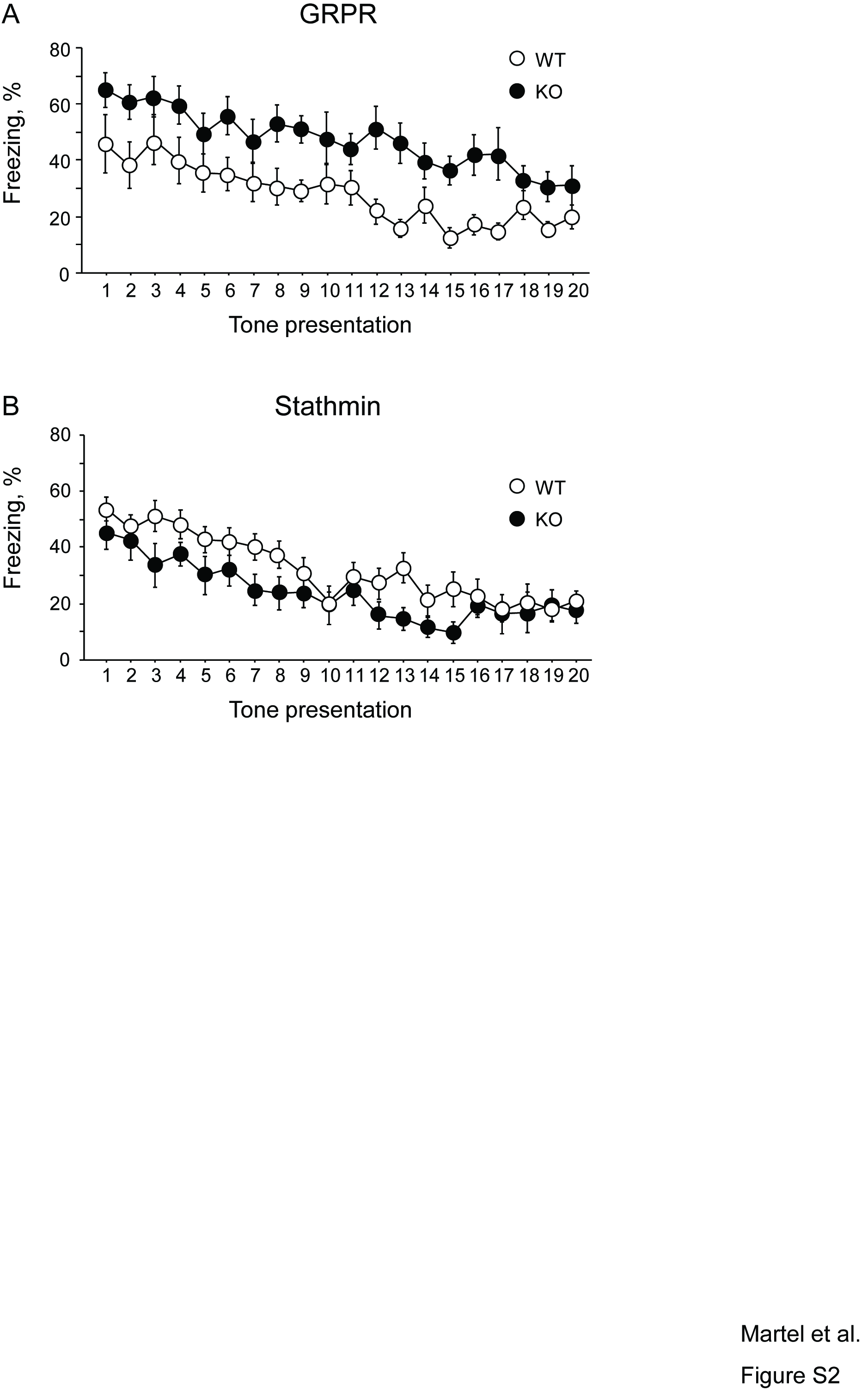

Supplement: Figure S2 — Analysis of the first day of cued extinction revealed that GRPR and stathmin KO mice started at the same level. (A) Percentage of freezing of GRPR KO mice during the first day of cued extinction. (B) Percentage of freezing of stathmin KO mice during the first day of cued extinction. Results are presented as mean ± SEM. (TIF) [file pone.0030942.s002.tif]

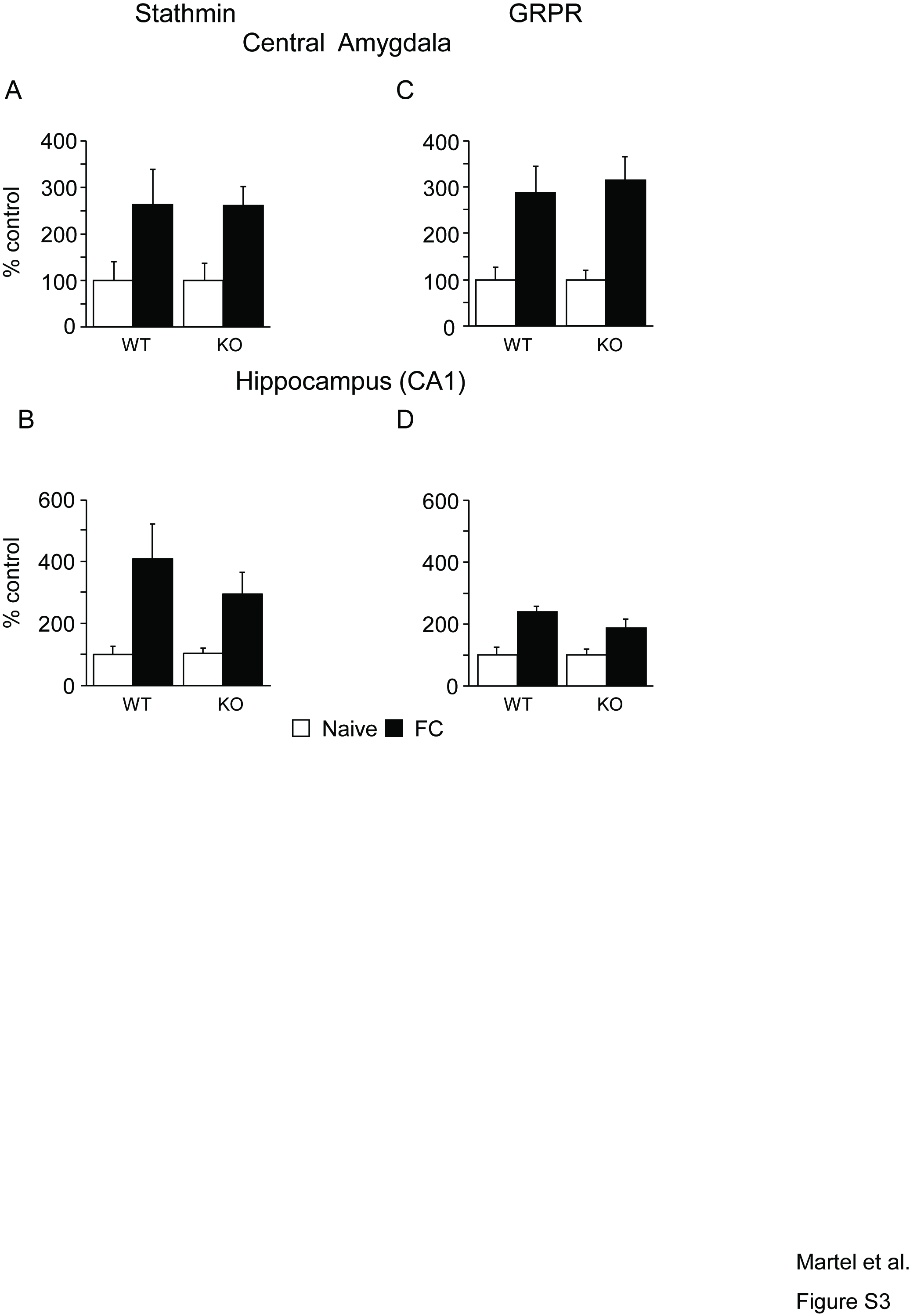

Supplement: Figure S3 — No difference in c-Fos induction in the central amygdala and CA1 area of hippocampus during extinction in stathmin KO and GRPR KO mice compared to wildtype controls. (A) and (C), c-Fos induction is the same in the central amygdala of stathmin KO and GRPR KO mice compared to their WT littermates. (B) and (D), c-Fos induction is the same in the CA1 hippocampal area of stathmin KO and GRPR KO mice compared to their WT littermates. Results are presented as mean ± SEM. (TIF) [file pone.0030942.s003.tif]

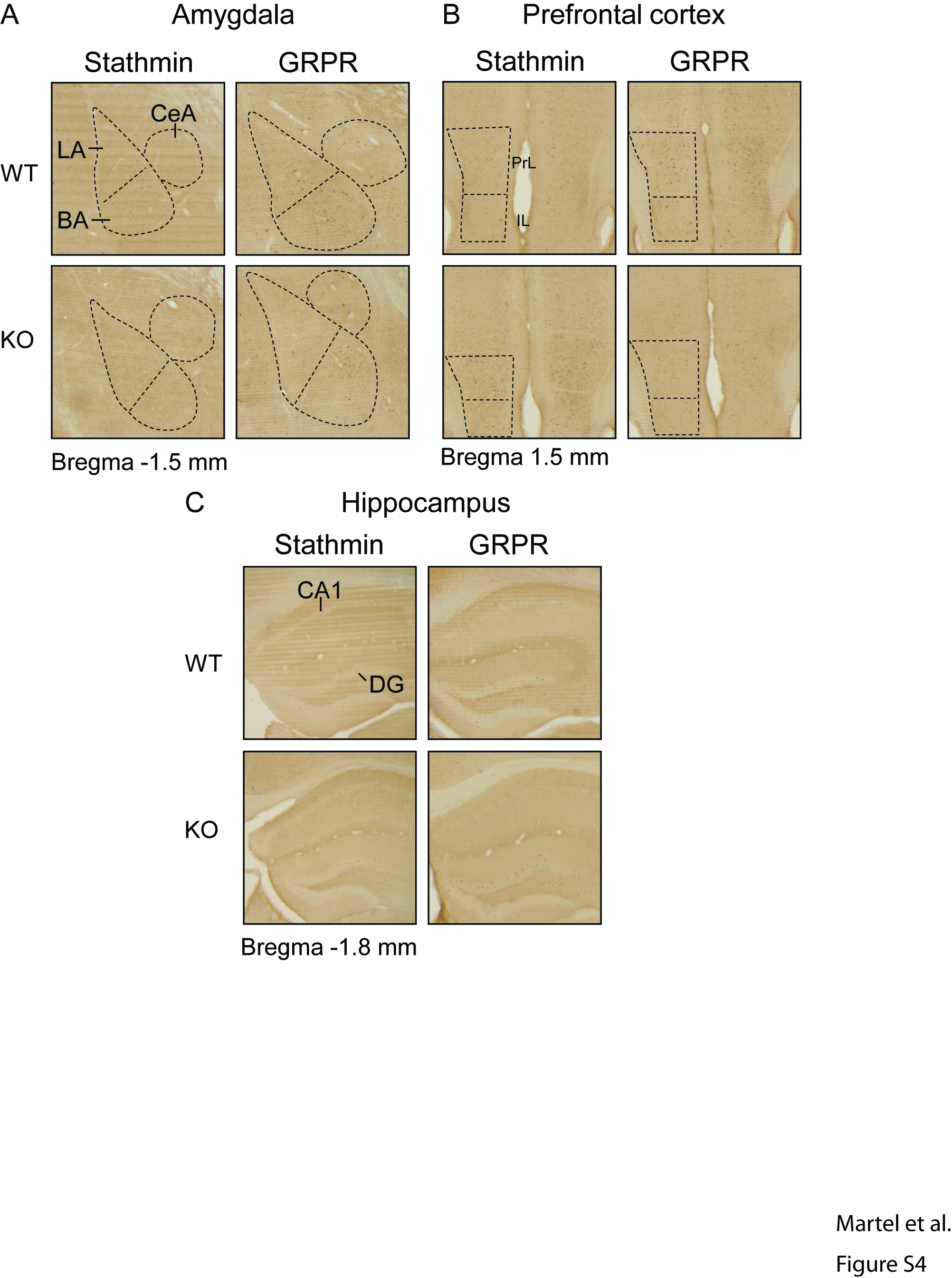

Supplement: Figure S4 — Representative photographs of c-Fos staining during extinction. c-Fos expression in the amygdala (A), prefrontal cortex (B) and hippocampus (C) of stathmin and GRPR WT and KO mice. (TIF) [file pone.0030942.s004.tif]
